# Addressing digital exclusion to improve access to HIV and viral hepatitis care for people who experience criminalization: a mixed methods evaluation of a quality improvement project

**DOI:** 10.1186/s12939-025-02648-3

**Published:** 2025-12-02

**Authors:** Amrit Tiwana, Nicola Gale, Mike Mahay, Tiffany Barker, Rebecca Hasdell, Pam Young, Mo Korchinski, Deb Schmitz, Daryl Luster, Alnoor Ramji, Julia MacIsaac, Brian Conway, Chris Fraser, Sofia Bartlett

**Affiliations:** 1 British Columbia Centre for Disease Control, Vancouver, BC Canada; 2 School of Community and Regional Planning, Vancouver, BC Canada; 3 CUPS Liver Clinic, Calgary, AB Canada; 4 Unlocking the Gates Services Society, Maple Ridge, BC Canada; 5 BC Hepatitis Network, Richmond, BC Canada; 6 St. Paul’s Hospital, Vancouver, BC Canada; 7 Division of Gastroenterology, Faculty of Medicine, University of British Columbia, Vancouver, BC Canada; 8 Department of Community Internal Medicine, Faculty of Medicine, University of British Columbia, Vancouver, BC Canada; 9 Vancouver Infectious Diseases Centre, Vancouver, BC Canada; 10 Faculty of Health Sciences, Simon Fraser University, Burnaby, BC Canada; 11 Cool Aid Community Health Centre, Victoria, BC Canada; 12 School of Population and Public Health, Faculty of Medicine, University of British Columbia, 2206 East Mall, Vancouver, BC V6T 1Z3 Canada

**Keywords:** Digital health equity, Hepatitis, HIV, Sexually transmitted bloodborne infections, Quality improvement

## Abstract

**Background:**

People who experience criminalization, such as those who use drugs, are incarcerated, and are affected by homelessness, have a high prevalence of HIV and/or hepatitis C virus (HCV) infection and low treatment uptake in British Columbia. Barriers to care include unreliable means of maintaining contact with healthcare providers. To reduce these barriers, the Test, Link, Call (TLC) Project provides cell phones and peer health mentors to support access to HIV and/or HCV care. This study aims to determine the outcomes and acceptability of TLC and its impact on care engagement.

**Methods:**

A mixed-methods evaluation was conducted over the first 29 months (October 2021–March 2024) of the TLC Project. Data were collected concurrently in two rounds: the first after one year and the second two years after launch. Qualitative data were collected using semi-structured interviews conducted with healthcare providers (*n* = 8), peer health mentors (*n* = 6), and program participants (*n* = 20). Quantitative data, including demographic and clinical information, were gathered through program records and cross-sectional clinical chart reviews. Factors associated with HCV treatment uptake were assessed among HCV RNA positive participants (*n* = 245) using multivariate logistic regression. Data from both rounds were integrated for comprehensive analysis.

**Results:**

273 participants were enrolled in HCV care, and 26 in HIV care. Interviewees found TLC highly acceptable and effective. Positive outcomes included increased access to health and social services, connection to loved ones, independence, and safety. Challenges included phone theft and digital literacy issues. Overall, 57% of TLC participants enrolled for HCV care initiated curative treatment, compared to 40% among people who currently inject drugs in the provincial administrative database in 2020. The multivariate logistic regression analysis suggested that gender, housing stability, safer supply prescriptions, and length of involvement in the TLC program are predictive factors influencing treatment initiation.

**Conclusions:**

The provision of cell phones and peer health mentors effectively increased engagement in HIV and HCV care, demonstrating substantial benefits despite some challenges. This cost-effective intervention could be expanded to support people who experience criminalization in other geographic locations and addressing other health conditions, such as syphilis and substance use disorder.

**Supplementary Information:**

The online version contains supplementary material available at 10.1186/s12939-025-02648-3.

## Background

People who experience criminalization (PWEC), including people who use drugs (PWUD), people with lived experience of incarceration (PWLE-I), sex workers, and those facing homelessness or unstable housing, have a high prevalence of sexually transmitted and bloodborne infections (STBBIs), such as human immunodeficiency virus **(**HIV), hepatitis C virus (HCV), and hepatitis B virus (HBV) [1–4]. These populations face significant barriers to healthcare, resulting in lower uptake of STBBI treatment than do those who do not experience criminalization [5, 6]. Limited access to a cell phone exacerbates these barriers [7]. Ensuring digital health equity (i.e., fair access to digital health services) is crucial for improving STBBI care, increasing engagement in treatment, and enhancing health outcomes.

In 2019, 1,958 new HCV cases were reported in British Columbia (BC), with 80% occurring among PWUD [8]. Similarly, in 2020, there were 108 new HIV cases, 23.2% of which were among PWUD [9]. These disparities are observed not only in BC [3–5, 10, 11] but also across Canada [2, 12–14], the United States [15, 16], and globally [1, 17, 18]. The largest gap in the 2020 BC Hepatitis Testers Cohort (BC-HTC) HCV care cascade is between the stages of diagnosis and starting curative treatment; while 73% of those with no history of intravenous drug use (IVDU) started treatment, only 40% started if they had current IVDU and 48% started if they had previous IVDU [5, 19]. Despite the availability of direct acting antiviral (DAA) medications, which can cure chronic HCV within 8–12 weeks with cure rates exceeding 95%, fewer than 15% of those with chronic HCV in BC had undergone treatment as of 2015 [20]. To achieve the World Health Organization’s 2030 target of 80% treatment initiation among diagnosed individuals [21], significant improvements in access to care are necessary.

The high prevalence of STBBIs among PWEC is driven by numerous barriers to care, including stigma [22–24], concerns about privacy [23, 24], and difficulties navigating the healthcare system [22, 23, 25]. Research has shown that stigma is often linked to social circumstances such as unstable housing and substance use rather than the STBBI diagnosis itself [22, 23]. PWEC report feeling distrusted by healthcare providers and face long delays between testing and treatment due to multiple required appointments, challenges with transportation, and digital connectivity issues, as healthcare providers may assume that patients will not follow up with them if they cannot connect with them [22]. In the BC correctional centers, some women living with HIV fear unauthorized disclosure of their status by staff [24]. Post release, securing basic necessities such as food and shelter often takes priority over health, complicating adherence to treatment [25].

Having a cell phone is a critical yet often overlooked barrier to healthcare access. Without a cell phone, individuals face significant barriers, such as the inability to search for health information, schedule appointments online, and participate in telemedicine consultations [26]. This digital gap exacerbates existing health disparities by hindering overall treatment and care. Addressing these challenges is essential, as digital tools and digital literacy are now recognized as critical determinants of health because of their connection to other upstream factors [27, 28]. Cell phone-based interventions offer promising avenues to enhance connections to healthcare services, particularly for STBBI care during post release periods for PWLE-I [26].

We adopted a version of the Dahlgren and Whitehead Socio-Ecological Model adapted to include the digital determinants of health [29] to address these barriers comprehensively. This model captures the relationships among digital determinants of health at the individual, social, and structural level. With the COVID-19 pandemic accelerating the shift to digital healthcare, access to digital tools has become increasingly important. This recent shift toward digital health has raised concerns about widening health inequities, prompting calls for inclusive strategies that mitigate disparities in access to digital tools [30, 31]. By focusing on digital health equity and bridging gaps between healthcare sectors (e.g., correctional facilities, acute care, housing, and primary care), we aim to reduce health disparities and improve STBBI care outcomes for high-risk populations, serving as a model for public health improvement and infectious disease burden reduction.

This paper describes the development, testing, and evaluation of the Test, Link, Call (TLC) Project, which is structured according to the SQUIRE guidelines [32]. Our objectives are to determine the outcomes and acceptability of TLC and evaluate its impact on engagement with HIV and HCV care among PWEC.

## Methods

### Setting and context

**Fig. 1:**
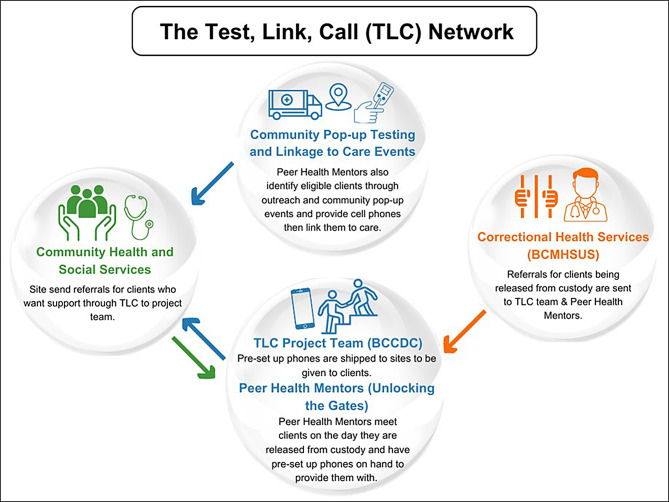
TLC Project Network: Collaborative partnerships for enhancing HIV and HCV access for PWEC

The TLC Project is an ongoing quality improvement (QI) initiative in BC, Canada, aimed at enhancing access to HIV and HCV care for PWEC. Launched in October 2021, it is a collaborative effort among the BC Centre for Disease Control, BC Mental Health and Substance Use Services, the BC Hepatitis Network, and Unlocking the Gates Services Society. This unique partnership includes government agencies, STBBI healthcare providers, prison health services, and peer organizations (Fig. 1).

Healthcare providers in correctional centers and the community, along with peer health mentors from Unlocking the Gates, receive training on recruitment, cell phone use, project specifics, and participant support. They identify eligible participants and invite them to join the TLC Project. Recruitment also occurs through posters in correctional centers, drug treatment programs, homeless shelters, and hospitals. Eligible participants are those who have experienced criminalization (e.g., PWUD, PWLE-I, sex workers, and those facing homelessness and unstable housing) and have disclosed an HIV or HCV infection to a peer health mentor or a healthcare provider. All diagnoses were confirmed through medical records or clinical testing (venous blood draw or dried blood spot). Individuals who self-reported a diagnosis that could not be confirmed through medical records or laboratory testing were deemed ineligible. Participants who consent to the project receive a free cell phone with a six-month unlimited talk and text plan, pre-installed with apps for overdose prevention, medication reminders, and mental wellness. They are also given the opportunity to connect to a peer health mentor who assists with accessing health and social services upon release from custody or in the community [33]. Participants receive the standard of care for their infection(s) from their associated healthcare clinic.

### Study design

A mixed methods approach using a transformative paradigm was used to evaluate the first 29 months (October 2021–March 2024) of the TLC Project. This approach focuses on the experiences of marginalized populations, analyzes power differentials, and links findings to actionable steps to reduce disparities [34]. The quantitative and qualitative data were collected concurrently in two rounds: the first after one year and the second two years after launch. Those who participated in the qualitative interviews were allowed to take part in both rounds of data collection, and their data were treated as separate observations rather than combined. By treating their data as separate observations, we avoid the assumption that responses from the same interviewee at different time points are identical or highly similar. This approach is especially important in capturing shifts in perceptions, experiences, or outcomes that may have occurred between rounds, providing a richer, more dynamic view of the project’s impact. Furthermore, since only a few interviewees (*n* = 2 healthcare providers and *n* = 2 peer navigators) took part in both rounds, combining their responses could inadvertently bias results or obscure nuanced changes that are essential to understanding the project’s effects over time.

The integration of quantitative and qualitative findings was a key part of the analysis and interpretation and was organized by the Dahlgren and Whitehead Socio-Ecological Model [29]. The qualitative findings were compared with patterns observed in the quantitative analyses to identify convergence (where results aligned), complementarity (where qualitative insights explained or expanded on quantitative patterns) and divergence (where results differed in ways that revealed gaps or inequities). By connecting the datasets through this model, we were able to triangulate and validate the findings across both data types, leading to a more comprehensive understanding of the barriers, facilitators, and outcomes within the TLC Project.

### Measures

We conducted semi-structured interviews with healthcare providers (*n* = 8), peer health mentors (*n* = 6), and project participants (*n* = 20) to understand their opinions and experiences with the project. A purposive sampling strategy was used to ensure representation from healthcare providers, peer health mentors, and participants across different stages of the care cascade. Recruitment continued until thematic saturation was reached, defined as no new themes emerging from interviews. Some individuals participated in both data collection periods, resulting in a total of 39 interviews. All of the interviews were conducted either over the phone or via Zoom and lasted between 30 and 60 min.

At the same time as the qualitative data collection, a data extraction template was sent to participating clinics and sites to gather clinical and demographic information. Partnering clinics used participant clinical charts to complete the data template and returned de-identified data to the TLC team. For participants enrolled in TLC, demographic information was also recorded at enrollment, with additional program data such as cell phone distribution tracked in program records. Retrospective chart reviews confirmed an undetectable HCV RNA result (i.e. sustained virologic response, or viral cure) at least 12 weeks after the completion of DAA treatment.

For participants enrolled for HCV care, demographic characteristics and comorbidity profiles are stratified by HCV care cascade stage and are described in Table 1. The stages of the HCV care cascade included: testing HCV RNA positive, completing treatment workup bloodwork, receiving a DAA prescription, starting treatment, completing treatment, and achieving an undetectable HCV viral load at 12 weeks post-treatment, or sustained virological response (SVR12), which indicates a viral cure. Demographic and clinical characteristics included: gender (man, woman, nonbinary), age (20–24, 25–29, 30–39, 40–59, 60+), housing status (no fixed address which includes sleeping primarily in shelters or “sleeping rough”/tenting/sleeping on the street; unstable housing which includes living in an SRO, “halfway house”, couch surfing, or detox/treatment facilities; incarcerated, housed, hospital, and stable housing), active IVDU if noted by clinician within 3 months of treatment workup (yes, no), Opioid Agonist Therapy (OAT; yes, no), safe supply prescription (yes, no), FIB-4 score (< 1.45, 1.45–3.25, > 3.25), cirrhosis (yes, no), treatment-naïve (yes, no), care type (peer-involved, clinic-involved only), and TLC enrollment date (October 2021–March 2022, April 2022–September 2022, October 2022–March 2023, April 2023–September 2023, October 2023–March 2024).

**Table 1:** Demographic characteristics and comorbidity profile of enrolled TLC participants, stratified by HCV care cascade stage

Variable *n* (row % of previous column)	Enrolled *n* (%)	HCV RNA Positive *n* (%)	Bloodwork done *n* (%)	Prescribed DAA *n* (%)	HCV Treatment initiated *n* (%)	HCV Treatment complete *n* (%)	SVR Achieved *n* (%)
Total	273 (100.00)	245 (89.47)	191 (78.00)	152 (79.58)	139 (91.45)	120 (86.27)	91 (75.83)
**Gender**							
Woman	91 (100.00)	78 (85.71)	59 (75.64)	39 (66.01)	36 (92.31)	29 (80.56)	23 (79.31)
Man	180 (100.00)	165 (91.67)	130 (78.79)	111 (85.38)	103 (92.79)	91 (88.35)	68 (74.73)
Non-Binary	2 (100.00)	2 (100.00)	2 (100.00)	2 (100.00)	0 (0.00)	0 (0.00)	0 (0.00)
**Age Cohort**							
20–24	2 (100.00)	2 (100.00)	2 (100.00)	2 (100.00)	1 (50.00)	1 (100.00)	1 (100.00)
25–29	11 (100.00)	10 (90.91)	7 (70.00)	4 (57.14)	4 (100.00)	2 (50.00)	0 (0.00)
30–39	71 (100.00)	60 (84.51)	45 (75.00)	34 (75.66)	31 (91.18)	24 (77.42)	15 (62.50)
40–59	155 (100.00)	141 (90.97)	109 (77.30)	86 (78.90)	79 (91.18)	71 (89.87)	56 (78.87)
60+	34 (100.00)	32 (94.12)	28 (87.50)	26 (92.86)	24 (92.31)	22 (91.67)	19 (86.36)
**Housing**							
No Fixed Address	89 (100.00)	74 (83.15)	61 (82.43)	41 (67.21)	39 (95.12)	36 (92.31)	27 (75.00)
Unstably Housed^1^	92 (100.00)	86 (93.48)	80 (93.02)	73 (91.25)	64 (87.67)	55 (85.94)	40 (72.73)
Incarcerated	11 (100.00)	10 (90.91)	10 (100.00)	6 (60.00)	6 (100.00)	5 (83.33)	3 (60.00)
Housed	20 (100.00)	19 (95.00)	18 (94.74)	17 (94.44)	16 (94.12)	13 (81.25)	12 (92.31)
Hospital	3 (100.00)	3 (100.00)	3 (100.00)	2 (66.67)	2 (100.00)	2 (100.00)	1 (50.00)
Unknown	58 (100.00)	53 (91.38)	19 (35.85)	13 (68.42)	12 (92.31)	9 (75.00)	8 (88.89)
**Active IVDU** ^2^							
Yes	84 (100.00)	70 (83.33)	63 (90.00)	47 (74.06)	43 (91.49)	37 (86.05)	28 (75.68)
No	31 (100.00)	30 (96.77)	28 (93.33)	27 (96.43)	24 (88.89)	20 (83.33)	14 (70.00)
Unknown	158 (100.00)	145 (91.77)	100 (68.97)	78 (78.00)	72 (92.31)	63 (87.50)	49 (77.78)
**On OAT**							
Yes	144 (100.00)	127 (88.19)	113 (88.98)	95 (84.07)	85 (89.47)	74 (87.06)	55 (74.32)
No	42 (100.00)	35 (83.33)	33 (94.29)	28 (84.85)	26 (92.86)	23 (88.46)	17 (73.91)
Unknown	87 (100.00)	83 (95.40)	45 (54.22)	29 (64.44)	28 (96.55)	23 (82.14)	19 (82.61)
**Prescribed Safer Supply**							
Yes	55 (100.00)	44 (80.00)	42 (95.45)	38 (90.48)	35 (92.11)	32 (91.43)	17 (53.13)
No	49 (100.00)	44 (89.80)	40 (90.91)	35 (87.50)	30 (85.71)	24 (80.00)	17 (70.83)
Unknown	169 (100.00)	157 (92.90)	109 (69.43)	79 (72.48)	74 (93.67)	64 (86.49)	57 (89.06)
**FIB-4 Score**							
<1.45	106 (100.00)	99 (93.40)	91 (91.92)	80 (87.91)	73 (91.25)	60 (82.19)	41 (68.33)
1.45–3.25	41 (100.00)	40 (97.56)	40 (100.00)	36 (90.00)	34 (94.44)	33 (97.06)	27 (81.82)
>3.25	5 (100.00)	5 (100.00)	5 (100.00)	5 (100.00)	4 (80.00)	3 (75.00)	3 (100.00)
Unknown	121 (100.00)	101 (83.47)	55 (54.46)	31 (56.36)	28 (90.32)	24 (85.71)	20 (83.33)
**Cirrhotic**							
Yes	11 (100.00)	10 (90.91)	10 (100.00)	10 (100.00)	8 (80.00)	6 (75.00)	6 (100.00)
No	148 (100.00)	134 (90.54)	126 (94.03)	110 (87.30)	102 (92.73)	88 (86.27)	63 (71.59)
Unknown	114 (100.00)	101 (88.60)	55 (54.46)	32 (58.18)	29 (90.63)	26 (89.66)	22 (84.62)
**Treatment Naive**							
Yes	84 (100.00)	71 (84.52)	68 (95.77)	59 (86.76)	51 (86.44)	43 (84.31)	27 (62.79)
No	25 (100.00)	21 (84.00)	20 (95.24)	18 (90.00)	17 (94.44)	16 (94.12)	13 (81.25)
Unknown	164 (100.00)	153 (93.29)	103 (67.32)	75 (72.82)	71 (94.67)	61 (85.92)	51 (83.61)
**Care Type**							
Peer-involved	114 (100.00)	106 (92.98)	83 (78.30)	62 (74.70)	56 (90.32)	46 (82.14)	39 (84.78)
Clinics only	159 (100.00)	139 (87.42)	108 (77.70)	90 (83.33)	83 (92.22)	74 (89.16)	52 (70.27)
**TLC Enrollment Date**							
Oct 2021–Mar 2022	15 (100.00)	12 (80.00)	10 (83.33)	8 (80.00)	8 (100.00)	6 (75.00)	6 (100.00)
Apr–Sept 2022	114 (100.00)	99 (86.84)	86 (86.87)	72 (83.72)	67 (93.06)	61 (91.04)	52 (85.25)
Oct 2022–Mar 2023	51 (100.00)	44 (86.27)	39 (88.64)	30 (76.92)	30 (100.00)	28 (93.33)	21 (75.00)
Apr–Sept 2023	27 (100.00)	26 (96.30)	22 (84.62)	15 (68.18)	11 (73.33)	7 (63.64)	5 (71.43)
Oct 2023–Mar 2024	66 (100.00)	64 (97.97)	34 (53.13)	27 (79.41)	23 (85.19)	18 (78.26)	7 (38.89)

Abbreviations: HCV, hepatitis c virus; OAT, opioid agonist therapy; IVDU, intravenous drug use; DAA, direct acting antiviral; SVR, sustained virologic response (i.e., viral cure); n, number

^1^Defined as living in an SRO, “halfway house”, couch surfing, or detox/treatment facilities

^2^Active IVDU, if noted by clinician within 3 months of treatment workup

As of August 2023, the TLC program enrollment criteria was expanded to include connection to HIV care. The demographic characteristics and comorbidity profiles of the HIV positive participants enrolled from August 2023–March 2024 are reported in Table 2.

**Table 2:** Demographic characteristics and comorbidity profile for HIV positive TLC participants

Characteristics *n* (%)	Overall *n* = 26
**Gender**	
Man	14 (53.85)
Woman	9 (34.62)
Non-Binary	3 (11.54)
**Age**	
20–39	8 (30.77)
40–59	10 (38.46)
60+	7 (26.92)
Unknown	1 (3.85)
**Housing**	
NFA	4 (15.38)
Unstably Housed	3 (11.54)
Housed	3 (11.54)
Unknown	16 (61.54)
**HIV VL**	
Suppressed^1^	9 (34.62)
Not Suppressed	3 (11.54)
Unknown	14 (53.85)
**CD4**	
> 200^2^	11 (42.31)
< 200	1 (3.85)
Unknown	14 (53.85)
**Enrollment Year**	
2023	21 (80.77)
2024	5 (19.23)

Abbreviations: HIV, human immunodeficiency virus; ARV, antiretroviral; VL, viral load; CD4, CD4 T lymphocytes

^1^Suppressed HIV viral load is defined as a viral load < 40 copies/mL, or < 200 copies/mL on a single occasion (i.e. a “blip”)

^2^Participants without severe immunosuppression, defined as participants with CD4 T lymphocyte counts above 200 cells/mm^3^

### Analysis

Qualitative analysis involved transcribing interviews verbatim and analyzing thematically using an inductive approach. Initial coding identified recurring themes related to the intervention and outcomes. Themes were categorized to understand barriers, facilitators, and overall experiences. Levesque’s Five As of Access (approachability, acceptability, availability, affordability, and appropriateness) were applied to interpret themes and link findings to actionable steps for improving STBBI care access [35]. Trustworthiness and validity checks, including interrater reliability, were used to ensure data quality and rigor.

Data on demographic, clinical, and program metrics were analyzed descriptively overall among the TLC project cohort that were enrolled for access to HCV (Table 1) and HIV care (Table 2). These variables were also stratified within each stage of the HCV care cascade to characterize the relationship to program outcomes (Table 1). To explore differences in care type, detailed demographic characteristics and comorbidity profiles stratified by peer-involved and clinic-only participants were also described (Supplementary Table 1). Factors associated with treatment uptake among HCV RNA positive TLC clients were assessed using multivariate logistic regression and corresponding 95% confidence intervals (CIs) (Table 3). All variables with a relaxed *P* value (*P* < 0.20) in the unadjusted univariate analyses were used in the final adjusted multivariate logistic regression model, with statistically significant differences assessed with a *P* value < 0.05. The adjusted analysis was limited by our small sample size, resulting in an events-per-variable (EPV) ratio of 50. However, previous research has found that an EPV of 50, 10, and even 2 [36–38] is sufficient enough for adequate estimation of regression coefficients, standard errors, and confidence intervals. Missing data were handled by retaining ‘unknown’ as its own category in the analysis. Given the high prevalence of unknown values across many variables, this approach was chosen to preserve the integrity of the data and avoid potential biases that could arise from excluding these cases. All analyses were conducted using STATA software.

**Table 3:** Logistic regression of factors associated with reaching the HCV care cascade “treatment start” stage

Variable *n* (%)^1^	Overall *N* = 245^a^	Unadjusted			Adjusted	
		OR (95% CI)	*P* value^b^		OR (95% CI)	*P* value
**Gender**						
Woman	78 (31.84)	Ref	–		Ref	–
Man	165 (67.35)	1.99 (1.15–3.43)	**0.014**		2.87 (1.29–6.42)	**0.01**
Non-Binary	2 (0.82)	–	–		–	–
**Age Cohort**						
30–39	60 (24.49)	Ref	–			
20–24	2 (0.82)	–	–			
25–29	10 (4.08)	0.62 (0.16–2.44)	0.49			
40–59	141 (57.55)	1.19 (0.65–2.18)	0.57			
60+	32 (13.06)	2.81 (1.09–7.23)	**0.033**			
**Housing Status**						
No Fixed Address	74 (30.20)	Ref	–		Ref	–
Unstably Housed	86 (35.10)	2.48 (1.27–4.81)	**0.008**		2.92 (1.27–6.68)	**0.011**
Incarcerated	10 (4.08)	1.28 (0.33–4.89)	0.72		–	–
Housed	19 (7.76)	4.53 (1.22–16.90)	**0.024**		11.5 (1.84–72.10)	**0.009**
Hospital	3 (1.22)	1.70 (0.15–19.60)	0.67		–	–
Unknown	53 (21.63)	0.25 (0.11–0.55)	**0.001**		–	–
**Active IVDU** ^2^						
Yes	70 (28.57)	Ref	–			
No	30 (12.24)	2.51 (0.91–6.94)	0.076			
Unknown	145 (59.18)	0.64 (0.36–1.14)	0.128			
**OAT**						
No	35 (14.29)	Ref	–			
Yes	127 (51.84)	0.73 (0.31–1.69)	0.457			
Unknown	83 (33.88)	0.18 (0.07–0.43)	**0.0**			
**Safer Supply**						
No	44 (17.96)	Ref	–		Ref	–
Yes	44 (17.96)	2.10 (0.78–5.68)	0.14		3.87 (1.03–14.6)	**0.046**
Unknown	157 (64.08)	0.42 (0.21–0.84)	**0.015**		–	–
**Cirrhotic**						
No	134 (54.69)	Ref	–		Ref	–
Yes	10 (4.08)	1.20 (0.24–5.97)	0.82		–	–
Unknown	101 (41.22)	0.12 (0.07–0.22)	**0.0**		0.12 (0.05–0.28)	**0.0**
**TLC Enrollment Date**						
Oct 2023–Mar 2024	64 (26.12)	Ref	–		Ref	–
Oct 2021–March 2022	12 (4.90)	3.33 (0.91–12.3)	0.07		5.86 (1.11–30.9)	**0.037**
Apr–Sept 2022	99 (40.41)	3.49 (1.81–6.74)	**0.0**		3.62 (1.37–9.53)	**0.009**
Oct 2022–Mar 2023	44 (17.96)	3.57 (1.59–8.04)	**0.002**		7.44 (2.25–24.6)	**0.001**
Apr–Sept 2023	26 (10.61)	1.22 (0.48–3.09)	0.67		–	–

Abbreviations: OR, odds ratio; CI, confidence interval; HCV, hepatitis c virus; OAT, opioid agonist therapy; IVDU, intravenous drug use; DAA, direct acting antiviral; n, number; CI, confidence interval

^a^HCV RNA positive clients enrolled in the TLC program

^b^Unadjusted variables with a p-value < 0.2 were included in the adjusted multivariate logistic regression

^1^Column percentages

^2^Active IVDU, if noted by clinician within 3 months of treatment workup

### Ethical considerations

The TLC Project was a QI project and did not require Research Ethics Board review as determined by the Provincial Services Health Authority (PHSA) Project Sorting Tool [39]. Instead, the project charter, materials, methods, and procedures were reviewed by the BC Mental Health and Substance Use Services Quality Committee, PHSA Risk Services, PHSA Legal Services, and the PHSA Office of Virtual Health to ensure adherence to relevant legislation and policies. For the interviews, all participants provided written informed consent. They were informed of their right to decline participation, withdraw at any time, and skip any questions they did not wish to answer. Peer health mentors and healthcare providers were also informed that their participation was voluntary and would not impact their professional responsibilities or program involvement. Project participants and peer health mentors who participated in interviews received a $25 cash payment for their participation. Healthcare providers employed by the health authority were not compensated. All data, including interview transcripts, clinical and demographic information was kept confidential. Interview participants were identified only by project numbers, with all personal identifiers, such as names and full dates of birth, removed. Payments to TLC project participants and peer health mentors were intended to recognize their time without creating undue influence and were consistent with provincial peer payment standards [40].

## Results

From October 2021–March 2024, 386 phones (including replacement phones) were given to eligible participants. During this period, the TLC project enrolled 273 participants for HCV care (Table 1). Most HCV participants were male (65.93%, 180/273), aged between 40 and 59 years (56.60%, 155/273), and either had no fixed address (32.60%, 89/273) or were unstably housed (33.70%, 92/273). Approximately one-third of the HCV participants had a known recent history of IVDU (30.77%, 84/273). Almost half had an unknown fibrosis stage using FIB-4 (44% of 121/273), while 69.7% (106/152) of FIB-4 scores were less than 1.45 suggesting unlikely to have extensive fibrosis. More than half of the HCV participants were on OAT (52.75%, 144/273). Only 4.03% (11/273) had cirrhosis by FIB-4. From August 2023–March 2024, the TLC enrollment criteria expanded to include access to HIV care, with 26 participants enrolled (Table 2). Most of the HIV participants were male (53.85%, 14/26), aged between 40 and 59 years (38.46%, 10/26), and had an unknown housing status (61.54%, 16/26). Most of the HIV participants had an unknown HIV viral load (53.85%, 14/26) and unknown CD4 count (53.8%, 14/26).

### Identified barriers in accessing STBBI care organized by the Dahlgren and whitehead socio-ecological Model

#### General socioeconomic, cultural, environmental, working, and living conditions

For many PWEC, factors affecting immediate survival often take precedence over STBBI treatment. A healthcare provider explained, “Hepatitis C sometimes is way on the back of the list. ‘I want to know where I’m eating today. I want housing. I don’t want to stay in the shelter anymore’” (Healthcare Provider 4, 2022). The lack of immediate symptoms from HCV makes it a low priority compared with other pressing health issues and daily challenges. One provider described the overwhelming nature of these challenges: “The simplest task feels like climbing Mount Everest sometimes when someone’s in that state” (Healthcare Provider 5, 2023).

Transportation issues further hinder access to STBBI care. A healthcare provider emphasized, “I think transportation is a huge issue. Getting out from Maple Ridge is a nightmare… When you’re looking at a three-hour bus ride, it’s pretty hard to convince somebody who would like treatment, but that’s not their top priority at the moment” (Healthcare Provider 4, 2022). Long commute times, lack of transportation, and limited availability of healthcare services in communities often discourage individuals from attending appointments. One participant expressed, “There’s nothing worse than having those appointments that you feel are so important and then being unsure if you’re gonna make it or not” (Participant 3, 2023).

The lack of cell phones among participants was a common barrier. One healthcare provider noted, “Most of our patients don’t have cell phones, and if they do, rarely do they have minutes for us to get in contact with them” (Healthcare Provider 5, 2022). The increasing reliance on telehealth in healthcare exacerbates this problem, with another provider stating, “So many clinics are not even offering in-person visits, so if you don’t have a phone, you can’t get healthcare” (Healthcare Provider 4, 2022).

#### Social and community networks

Fear and stigma further complicate matters, discouraging PWEC from seeking STBBI care. A peer health mentor explained, “It can be really stigmatizing to go to the doctor, especially if someone’s actively using. There’s a fear of who they’re actually going to see and what they’re going to be like” (Peer Health Mentor 1, 2022). Another mentor shared, “There’s a lot of fear around the treatment and being sick” (Peer Health Mentor 3, 2023). This fear often stems from negative past experiences with medication or perceptions of judgment within healthcare settings.

The lack of a support network complicates access to STBBI care, particularly for PWEC who struggle with navigating the healthcare system. A healthcare provider described, “A lot of our clients are not attached to anybody, so they don’t know what to do or who to talk to” (Healthcare Provider 2, 2023). The participants also highlighted the barrier of insufficient information about available services, with one noting, “Just not knowing how or where to access it” (Participant 4, 2022). A peer health mentor emphasized the difficulty in finding healthcare providers and starting treatment: “It’s so hard to find a doctor, let alone hep C treatment. It’s daunting and terrifying for many to even begin the process” (Peer Health Mentor 3, 2022).

### TLC project outcomes

All interviewees emphasized the positive impact of cell phones in facilitating communication and motivating the initiation of STBBI care. One participant noted, “It’s made a great big difference considering I would never have been able to make any of my appointments not having a phone. I wouldn’t be able to do any of the things I’m doing now without a phone” (Participant 7, 2023). Healthcare providers echoed this sentiment. One stated, “The cell phone is quite a big motivator for people to start treatment … Nobody likes getting their blood work done, so the phone is a great incentive for some clients to get it done” (Healthcare Provider 3, 2023). Another participant appreciated the convenience of virtual consultations, stating, “It’s just a phone call away now whereas before I used to have to go in face to face. It’s different now. It’s much better. That’s the part I’m enjoying” (Participant 2, 2022).

Peer support played a critical role in facilitating access to care in the project. Healthcare providers and mentors emphasized the importance of nonjudgmental support. One healthcare provider highlighted this by stating, “I think by having someone like me or a peer support worker … who understands and is willing to just sit there through the process and just walk beside them” (Healthcare Provider 5, 2023). A mentor echoed this sentiment: “They say, ‘I’ve tried to sign up for getting treatment for my hepatitis, but it never happens. Either the healthcare providers never bothered to follow us through, or other people didn’t really want to help.’ So, I say, well, there’s always somebody here who’s going to help you” (Peer Health Mentor 5, 2023). The participants also highlighted the value of peer support, with one stating, “Any other time I would make an appointment and never show up. So, with having the peer health mentor and the phone, I can keep accountable” (Participant 3, 2022). Mentors often share their personal experiences, fostering deeper connections with participants. As one mentor explained, “Because I have that shared lived experience, I kind of connect with people on like a deeper level than maybe just your average person. I like being able to share my experience and being able to share like what worked for me over the years” (Peer Health Mentor 1, 2023).

**Fig. 2:**
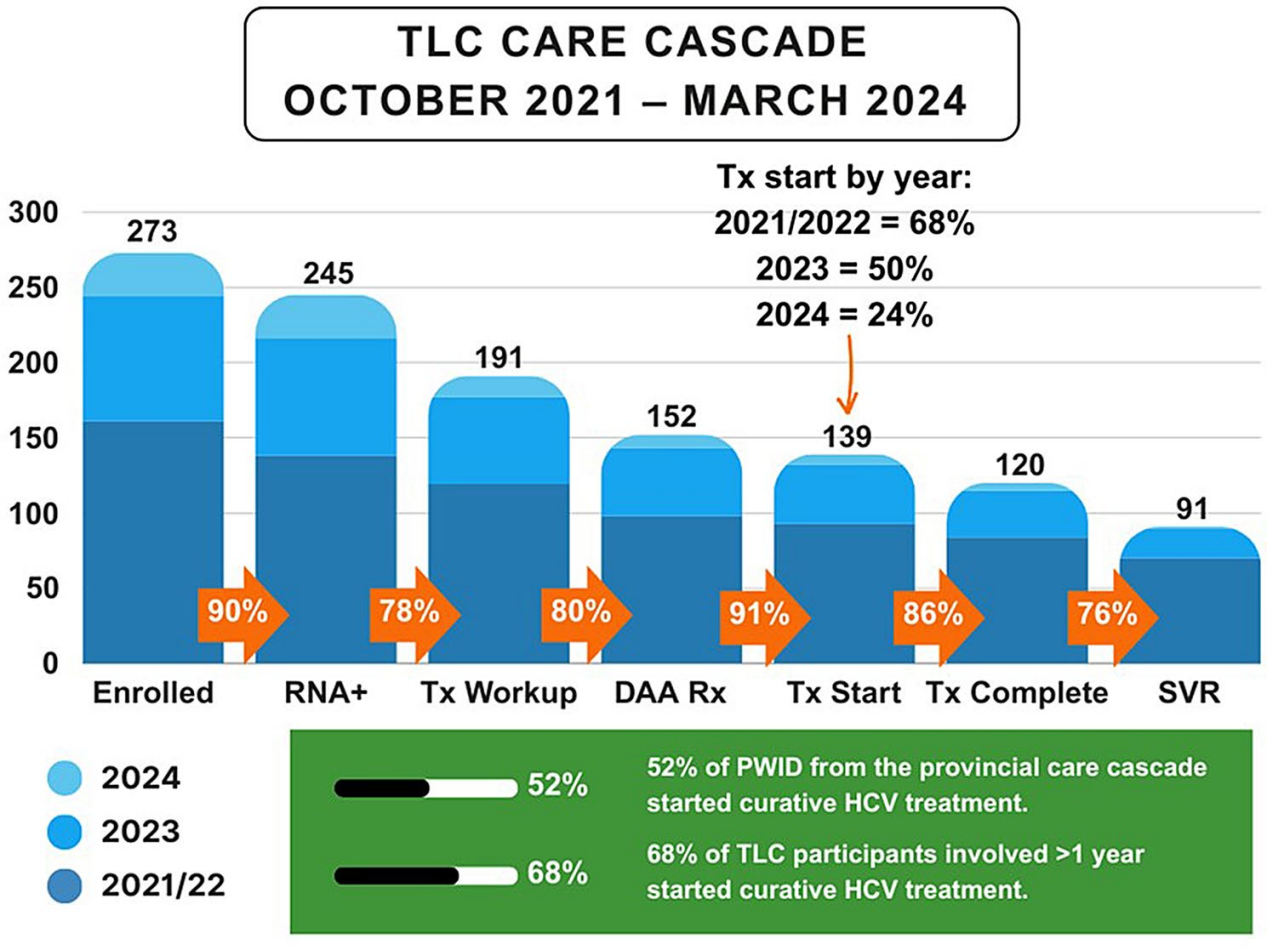
Progression in HCV care cascade among TLC project participants enrolled from October 2021–March 2024

Overall, 90.11% (245/273) of participants enrolled for HCV care tested RNA positive, 77.96% (191/245) of participants completed the necessary bloodwork, 79.58% (152/191) received a DAA prescription, 91.45% (139/152) started DAA treatment, 86.33% (120/139) completed DAA treatment. Among people who were tested for HCV RNA at least 12 weeks after completing HCV treatment, 100% of them achieved SVR12, however only 75.83% (91/120) of people who completed DAA treatment had an SVR12 test completed (Fig. 2). Overall, 56.73% (139/245) of all HCV RNA positive participants enrolled in the TLC program started curative treatment as of March 2024. When stratified by time elapsed since program enrollment, this increased to 67.74% (105/155) for participants enrolled longer than one year (before April 2023): 66.67% (8/12) of participants enrolled from October 2021–March 2022, 67.78% (67/99) of participants enrolled from April-September 2022, and 68.18% (30/44) of participants enrolled from October 2022–March 2023 started on DAA treatment. Treatment start decreased for participants enrolled for less than one year: 42.31% (11/26) of participants enrolled between April 2023–September 2023, and to 35.94% (23/64) of participants enrolled between October 2023–March 2024 were started on DAA treatment.

In the unadjusted logistic regression model of factors associated with DAA treatment start, the variables significant at a *P* < 0.2 level and included in the multivariate adjusted analysis were gender, age cohort, housing status, use of OAT, use of safer supply prescriptions, cirrhosis, and TLC enrollment date (Table 3). The multivariate logistic regression analysis demonstrated that men had almost three times the odds of starting curative DAA treatment than women did (adjusted odds ratio [aOR] 2.87; 95% CI 1.29–6.42). Participants with unstable housing (aOR 2.92; 95% CI 1.27–6.68) and stable housing (aOR 11.5; 95% CI 1.84–72.1) had higher odds of starting treatment compared to those with no fixed address. TLC participants with safer supply prescriptions had almost four times the odds of starting treatment (aOR 3.87; 95% CI 1.03–14.6) compared to those without. Additionally, participants enrolled between October 2021–March 2022 (aOR 5.86; 95% CI 1.11–30.9), April–September 2022 (aOR 3.62; 95% CI 1.37–9.53), and October 2022–March 2023 (aOR 7.44; 95% CI 2.25–24.6) had higher odds of starting treatment than did those enrolled between October 2023–March 2024. There was weak evidence of an association between starting DAA treatment and age, active IVDU, using OAT, and having cirrhosis.

### Limitations and challenges

Some participants faced challenges with phone security, often resulting in anxiety and having to take precautionary measures. A mentor noted the prevalence of phone theft, saying, “I think it’s sad that a lot of them have their phones stolen … they have to pretty much attach it to their pants you know and their crotch because it’s a hot commodity on the streets.” (Peer Health Mentor 2, 2023). Another provider shared the extreme caution of a participant living in a shelter, “He’s terrified of getting it stolen because he is in a shelter. He’s asked the outreach team to hold on to it until he’s in a more stable housing, and so he checks it periodically. It’s not great ‘cause you still don’t talk to him.” (Healthcare Provider 4, 2022). One participant expressed their strategy to avoid theft, “I leave it at home most of the time. I don’t like walking around with it. It’s the easiest little thing somebody could take.” (Participant 3, 2023). Another participant echoed this sentiment, stating, “I keep it close to me … I don’t let my eyes off of it. It stays on me all the time.” (Participant 2, 2022). These experiences, along with the loss of 86 phones, highlight the persistent issue of phone security, which affects participants’ ability to fully utilize their cell phones.

Digital literacy emerged as both a limitation and an opportunity for participants. One participant expressed a desire to explore more phone capabilities: “The only challenge is that I want to learn more about the phone. Like what I can do with it. Maybe I could do a job through my phone. I just want to find out what else is there on this phone that would be beneficial to me, you know but I don’t know” (Participant 4, 2023). The same participant, reflecting on their limited digital skills, said: “Still, I’m a baby learning because I don’t do Facebook. I don’t do Wi-Fi. I don’t do chat online or chat talk. I would love to learn how to explore doing that” (Participant 4, 2023). Another participant acknowledged being new to digital technology but was eager to learn: “I’m just starting to see what this is for, what that’s for. I’m starting to read about it and everything. Yeah, I’ve been checking out the apps” (Participant 2, 2022). The participants also found the phone useful for educational purposes: “I also look up information for communicable diseases and stuff” (Participant 1, 2023). Despite these challenges, the phone provided a gateway to digital learning and access to valuable information.

### Unintended benefits

The TLC Project improved connections far beyond STBBI care, including links to loved ones, other health services, housing, and jobs. A healthcare provider noted the increased connection to family members: “One of our clients was pretty excited that he could actually phone his son because he’s always switching phones, switching numbers, switching minutes. When I asked him how this phone helped you, he said, ‘well, I can call my son when I want to now’” (Healthcare Provider 5, 2022). A participant mentioned using the phone to address other health issues: “I got to use the phone to contact the optometrist and the dentist. My teeth are going bad on me because I have been smoking crystal meth and it’s doing a number on my teeth, so I made an appointment with the dentist and got in touch with them. And I wear glasses so I’m trying to get new glasses again” (Participant 2, 2023). Mental health also improved, as one participant mentioned, “Everything’s way better with my phone… because of my mental health, it’s easy for me to block something out and just stay focused on my phone and play games and stuff” (Participant 2, 2023). Another participant highlighted the improved convenience in accessing housing and job opportunities with the phone: “I was able to use that phone and make phone calls, read messages, leave contact numbers for all the people that I was trying to contact for housing and for jobs. I want to get back into the working field. I was calling different various jobs that I used to work for” (Participant 4, 2023).

The TLC Project significantly increased independence and safety. The participants expressed frustration with the lack of public pay phones, with one stating, “There’s no payphones anymore, and you can’t just ask someone to use their phone” (Participant 11, 2023) A peer health mentor highlighted the benefits of having a phone: “Oh, it’s a huge difference because you know, going from not having any way to communicate… there’s no pay phones anymore. People are very unlikely to loan their phone out… so I think having the phone gives them a sense of belonging and a sense of pride” (Peer Health Mentor 3, 2022). A healthcare provider noted, “It gives them a little bit of independence because now they don’t need to go to a pharmacy to ask them if they can use the phone, which during COVID was so hard” (Healthcare Provider 2, 2023). Safety was another benefit, with a participant saying, “Having a phone, it makes you feel a heck of a lot safer too!” (Participant 2, 2022) and another adding, “It’s nice to have the phone because then I can be like ‘Please if you don’t leave me alone then I’ll phone the police on you.’ And then they speed off, so it helps with safety for sure” (Participant 2, 2023).

The TLC Project effectively addressed barriers to healthcare access across multiple layers of the Dahlgren and Whitehead Socio-Ecological Model. One participant shared, “It makes me feel good to have the phone and have these people that are helping me” (Participant 2, 2022). A healthcare provider noted, “Most people, they just light up when they get the phone; it’s like the nicest thing they’ve had in a long time” (Healthcare Provider 1, 2023). The project also served as a bridge to broader healthcare and social services, encouraging participants to seek out additional services beyond STBBI care. A peer noted, “It’s not just about hepatitis C; it’s also a chance to bridge, you know, ‘Have you had a mammogram, have you had your pap smear, have you had colon testing?‘” (Peer Health Mentor 4, 2023), highlighting the trust built through positive healthcare experiences. One participant shared, “This phone has helped me amazingly. I have an intense case management team and they get a hold of me through this phone” (Participant 11, 2022). Another participant emphasized the phone’s role in managing legal responsibilities, stating, “I’m using it to stay on top of my bail appointments and stuff like that. My probation officer calls me because I have to get letters of permission to go out. So it’s good to have a phone to contact my bail supervisor and keep up with these appointments. It really helps out all around” (Participant 7, 2022). These experiences demonstrate the project’s holistic approach. As one peer explained, “We can’t separate just TLC, you know, hepatitis C out of the person because there’s so many more issues… we’re also about addiction, homelessness, mental health… it all goes together”. The TLC Project’s comprehensive approach not only improved participants’ immediate health outcomes but also fostered trust and engagement, encouraging a more holistic and continuous relationship with healthcare services.

## Discussion

### Summary

This study used a mixed methods approach to determine the outcomes and acceptability of the TLC Project and evaluate its impact on engagement with HIV and HCV care among PWEC. By providing a free cell phone with a six-month unlimited talk and text plan along with the support of a peer health mentor, the TLC Project significantly improved participants’ engagement with STBBI care. Despite challenges such as phone security and digital literacy issues, the project offered valuable learning opportunities for using a phone, strengthened connections with loved ones and other healthcare services, and promoted independence and safety. These findings contribute to the limited published literature on the evaluation of cell phone-based interventions aimed at improving digital health equity and healthcare access among marginalized populations and provides an applied example of how digital interventions operate through pathways in the Dahlgren and Whitehead Socio-Ecological Model.

A key strength of the TLC program is its unique multidisciplinary partnership between correctional facilities, acute care, housing, and primary care, which effectively reduces disparities and improves STBBI care outcomes for high-risk populations. Unlike the fragmented Canadian healthcare system, where patient care is often siloed across multiple independent organizations [41], this program fosters collaboration across healthcare, substance use, HIV and viral hepatitis care, and post-prison support services. Furthermore, the program’s evaluation is also strong due to its mixed-method approach, which combines quantitative and qualitative data to provide a comprehensive understanding of the program’s impact. The use of external researchers to evaluate the project ensures an unbiased assessment and having two researchers analyze the data further enhances the reliability and validity of the findings.

### Interpretation

The TLC Project’s intervention was successful in improving engagement in the care cascade for participants living with HCV. In our project, 57% of all TLC participants enrolled for HCV were started on curative treatment, compared to 40% of people who currently inject drugs identified in the BC-HTC [5, 18]. When stratified by year, treatment uptake increased up to 67.74% (105/155) for people involved in the TLC Project for over one year. Integrating these findings with the qualitative data, this increase in treatment uptake is likely due to enhanced communication capabilities offered by the phones and the continuous social support from peer health mentors. While timely access to cure is an important outcome, the length of time between enrollment and treatment start highlights the necessity of long-term engagement and holistic social support in overcoming barriers to care. It may also demonstrate the time required to connect participants to healthcare in general, income supports, and government services including housing waitlists, many of which can be hard to obtain without the system navigation and advocacy skills of the peer health mentors. This is reflected in another study which demonstrated that providing consistent, long-term support through peer mentors plays a significant role in facilitating health engagement [42]. Another study revealed that patients living with HIV were more likely to have appointments if providers treated them with dignity and respect, listened carefully to them, explained in ways that they could understand, and knew them as persons [43].

The TLC Project has a significant impact on participants, leading to increased independence, safety, and strengthened connections with loved ones. This aligns with findings from other studies highlighting the importance of digital inclusion for health outcomes. For instance, a study emphasized that digital exclusion, particularly among individuals with severe mental illness, negatively impacted health by reducing social connectedness and empowerment and revealed that key facilitators for increasing engagement included access to digital tools, digitally engaged social support networks, and personalized and straightforward digital tools [44]. Another study revealed that social isolation is a risk factor for poor health and increased mortality among people living with HIV [45]. The positive personal impacts observed in the TLC Project are similar to those seen in the PHONE-CONNECT Program in Ontario, which provides donated cell phones to digitally underserved patients discharged from the emergency department [46]. These programs highlight the importance of digital tools in enhancing social connectedness and community networks which are a well-known social determinant of health.

**Fig. 3:**
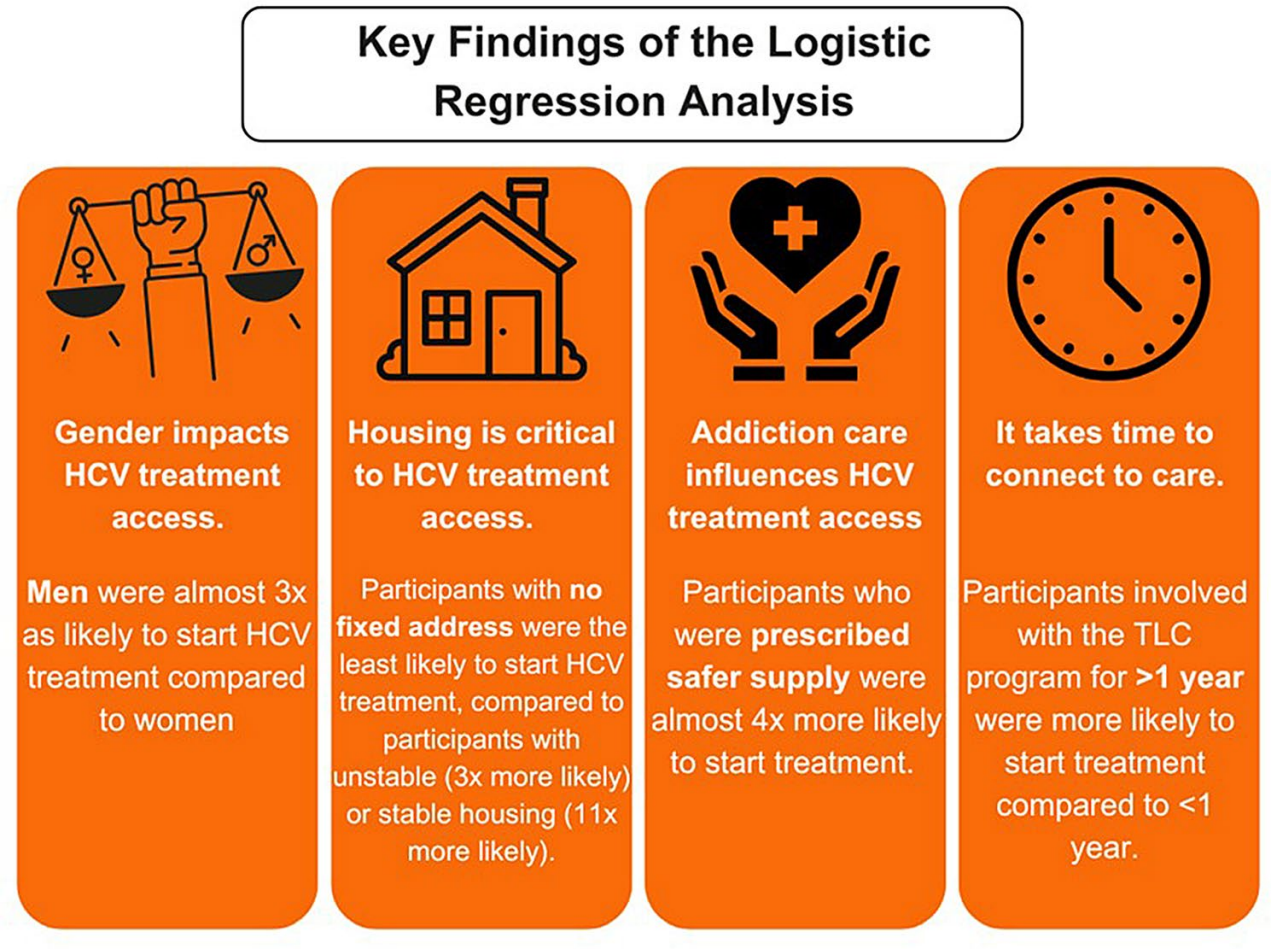
Key findings of the multivariate logistic regression analysis for the TLC project

Our findings indicate that gender, housing stability, and access to safer supply prescriptions significantly influence treatment uptake (Fig. 3). Men were found to have higher odds of starting treatment compared to women, suggesting potential differences in access to or engagement with care based on gender. This finding is echoed in an analysis of the BC-HTC study that found that women in BC experiencing poverty, social isolation, and problematic substance use experience gaps in access to HCV care compared to men [47]. Housing stability also plays a critical role in access to cure, as TLC participants with unstable or stable housing were significantly more likely to initiate HCV treatment compared to those with no fixed address, or who are “sleeping rough” or in shelters. This finding aligns with a study that examined factors associated with HCV treatment uptake among marginalized patients participating in psycho-educational support groups and found stable housing was independently associated with the initiation of treatment [48]. Access to safer supply prescriptions was also significantly associated with HCV treatment uptake, which is congruent with reports from across Canada that note higher engagement in care, including HCV and HIV treatment, through involvement with safer supply programs [49]. These results suggest that targeting socioeconomic conditions, improving housing access and stability, adapting programming to accommodate women’s needs, and expanding safer supply programs could be effective strategies to enhance STBBI treatment uptake and improve health outcomes within this population.

Our findings also indicate that the length of involvement in the program significantly influences treatment uptake. Participants involved longer with the TLC project were more likely to initiate treatment, suggesting that treatment engagement, workup, and initiation can often take longer than a year. This suggests that the current six-month timeframe for providing cell phones and minutes may be insufficient for many to reach the treatment start stage of the HCV care cascade. In addition, the odds of starting treatment increased from October 2021–March 2022 to October 2022–March 2023, during which several project improvements were implemented, including the distribution of phone cases, Indigenous medicine bundles, updated participant handbook about the phone, and enhanced recruitment strategies. Future projects should consider extending the treatment period beyond six months and continuing to refine project administration and recruitment efforts to improve completion rates and overall effectiveness.

Overall, the outcomes from this evaluation demonstrate the value of mixed methods analyses that pair qualitative and quantitative data to describe the challenges in accessing STBBI care, as well as access to health and social care more generally. Many of the barriers identified in the qualitative interviews highlighted the socioeconomic deprivation experienced by TLC participants (lack of stable housing, social connectivity, transportation options, stigma, etc.), which are not reflected in the biomedical model of the HCV care cascade. At face value, the movement of participants from the stages of HCV diagnosis to treatment start represents a linear step. However, the qualitative interviews demonstrate the uphill battle fought by participants and peers to address immediate survival needs before they can walk through the doors of a clinic. This highlights the utility of contextualizing biomedical models of care within a socioecological lens that can more ably stress the “experiential, interactive, and dynamic nature of negotiating access to care” and that incorporate a patient’s holistic and lived experience [50]. Considering marginalized and highly stigmatized communities experience a disproportionate burden of STBBIs, utilizing only a narrow view of program success that is primarily focused on quantitative results loses sight of the larger structural, political, and social determinants of health that hinder access to not only STBBI care but primary care, housing, and the basic necessities of life.

Despite challenges such as phone theft and digital literacy issues, the project’s long-term benefits and potential healthcare savings outweigh the costs, as shown in the TLC Project Impact Evaluation Report 2021–2022 [51]. The financial burden on patients with chronic illnesses, such as HCV, has been found to be significant, encompassing out-of-pocket costs and time costs for both patients and caregivers [52]. Balancing immediate costs with long-term healthcare improvements presents a strategic trade-off that highlights the value of such interventions.

### Limitations

There are some limitations of the program worth mentioning. The provision of phones to PWEC makes it vulnerable to theft, and the underlying poverty issues mean it serves as a temporary solution without addressing deeper inequities related to substance use and poverty. Additionally, its effectiveness is limited by digital literacy issues, an aging homeless population, and cognitive impairments. Feedback from healthcare providers and participants highlights the difficulties older participants face in adapting to this model. To mitigate these challenges, improvements such as providing phone cases, updating pamphlets with clear information about phone use, and offering targeted support have been implemented to enhance accessibility and usability. With these enhancements, we anticipate seeing higher treatment and completion rates in future rounds of data collection.

The qualitative evaluation has limitations, including narrowly focused research questions that restricted the exploration of perceptions and experiences across different subpopulations affected by criminalization. To address this, efforts were made to incorporate feedback loops with participants and stakeholders during the first data collection round to refine and expand the scope of inquiry where possible. Additionally, the assessment did not fully explore additional benefits, such as impacts on housing or overdose prevention, due to the data collection and evaluation plan’s focus. Future evaluations should consider a more inclusive sampling strategy to capture diverse perspectives.

Quantitative data limitations include the number of unknown observations, ranging from 20–61% for some variables. Instead of treating these as missing data, we included ‘unknown’ as its own category in the analysis. While this approach preserves data integrity and reflects the full scope of the data collected, it may limit statistical power and introduce potential bias. The majority of unknown data came from early in the TLC program rollout and from participants involved with peers (who do not have access to some clinical and demographic data), with data collection improving over the course of the evaluation collection period. Additionally, much of the clinical data for participants receiving HIV care through the project were unknown because their enrollment was recent, and no information had yet been received from partnering clinics. Furthermore, this evaluation was not designed to assess the secondary outcomes identified in the logistic regression analysis, and these results should therefore be considered as exploratory and indicative of areas for further investigation and program improvement. We anticipate that future rounds of data collection will have fewer missing unknowns, larger sample sizes, and longer follow up periods. A new platform in development, the Bridge2care app, is anticipated to improve data quality as information is collected directly from participants rather than indirectly through their healthcare providers.

### Implications

The findings from the TLC Project demonstrate the potential of integrating digital tools with personalized support to enhance healthcare access and outcomes among marginalized populations. This approach not only addresses immediate healthcare needs but also contributes to broader efforts to reduce health disparities and promote digital health equity. Policymakers and healthcare providers could consider adopting similar models to improve engagement in care for other chronic conditions or in different geographic settings. Additionally, these findings highlight the importance of addressing barriers such as digital literacy and phone security to maximize the impact of such interventions. Future initiatives could benefit from incorporating lessons learned from the TLC Project to design more inclusive and effective programs, potentially expanding to other regions or populations to further bridge gaps in healthcare access.

## Conclusion

The evaluation of the TLC Project demonstrated improved access to HIV and/or HCV care for PWEC by providing a free cell phone and peer support mentorship. This intervention addresses the barrier of limited access to digital tools, leading to higher rates of treatment initiation and completion, compared to standard care. TLC participants reported significant benefits, including better communication and easier access to health and social services. Peer mentors help foster trust and guide participants through the healthcare system. The TLC Project’s approach highlights the potential of such interventions to bridge gaps in healthcare access and reduce health disparities. Expanding similar initiatives for different health conditions, such as syphilis and substance use disorder, and in other geographic locations could further enhance care for marginalized populations and meet global health targets.

## Supplementary Information

Below is the link to the electronic supplementary material.

Supplementary Material 1

## Data Availability

The data used during the current study are available from the corresponding author upon reasonable request.

## Declarations

### Author information (optional)

Dr. Sofia Bartlett (she/her) is the senior author of this research article and currently serves as the Interim Scientific Director for Sexually Transmitted and Blood-Borne Infections at the BC Centre for Disease Control within Clinical Prevention Services. Her research focuses primarily on hepatitis C virus (HCV) and improving health outcomes for marginalized populations, including people who use drugs, those who are incarcerated, and individuals experiencing unstable housing. Dr. Bartlett is the Principal Investigator of the Test, Link, Call (TLC) project, where she leads efforts to facilitate HCV treatment referrals to clinics with experience in serving incarcerated individuals. As the Project Lead for TLC, she provides cell phones to project recruiters, supports recruitment activities, and helps monitor clients’ treatment progress. To learn more about Dr. Bartlett and her work, please visit stbbipathways.ca.
