# Supplementary material for: Addressing digital exclusion to improve access to HIV and viral hepatitis care for people who experience criminalization: a mixed methods evaluation of a quality improvement project
Source: Int J Equity Health. 2025 Dec 2;24:337. doi: 10.1186/s12939-025-02648-3 (PMC12673783; doi:10.1186/s12939-025-02648-3)
Supplement: Supplementary file 1 — Supplementary Material 1 [file 12939_2025_2648_MOESM1_ESM.docx]

**Supplementary Table 1** Demographic characteristics and comorbidity profile for peers- and clinic-involved TLC participants.

| Characteristics  n (%) | Peer-Involved  n = 114 | Clinic Only  n = 159 |
| --- | --- | --- |
| Gender |  |  |
| Man | 79 (69.30) | 101 (63.52) |
| Woman | 35 (30.70) | 56 (35.22) |
| Non-Binary | 0 (0.00) | 2 (1.26) |
| Age |  |  |
| 20-29 | 5 (4.39) | 8 (5.03) |
| 30-39 | 36 (31.58) | 35 (22.02) |
| 40-59 | 60 (52.63) | 95 (59.72) |
| 60+ | 13 (11.40) | 21 (13.21) |
| Housing |  |  |
| No Fixed Address | 41 (35.96) | 48 (30.19) |
| Unstably Housed | 28 (24.56) | 64 (40.25) |
| Incarcerated | 4 (3.51) | 7 (4.40) |
| Housed | 7 (6.14) | 13 (8.18) |
| Hospital | 0 (0.00) | 3 (1.89) |
| Unknown | 34 (29.82) | 24 (15.09) |
| IVDU^1^ |  |  |
| Yes | 25 (21.93) | 59 (37.11) |
| No | 11 (9.65) | 20 (12.58) |
| Unknown | 78 (68.42) | 80 (50.31) |
| OAT |  |  |
| Yes | 46 (40.35) | 98 (61.64) |
| No | 13 (11.40) | 29 (18.24) |
| Unknown | 55 (48.25) | 32 (20.13) |
| Safer Supply |  |  |
| Yes | 9 (7.89) | 46 (28.93) |
| No | 18 (15.79) | 31 (19.50) |
| Unknown | 87 (76.32) | 82 (51.57) |
| FIB-4 Score |  |  |
| <1.45 | 41 (35.96) | 65 (40.89) |
| 1.45-3.25 | 11 (9.65) | 30 (18.87) |
| >3.25 | 2 (1.75) | 3 (1.89) |
| Unknown | 60 (52.63) | 61 (38.44) |
| Cirrhotic |  |  |
| Yes | 8 (7.02) | 3 (1.89) |
| No | 47 (41.23) | 101 (63.53) |
| Unknown | 59 (51.75) | 55 (34.59) |
| Treatment Naive |  |  |
| Yes | 16 (14.04) | 68 (42.76) |
| No | 6 (5.26) | 19 (11.95) |
| Unknown | 92 (80.70) | 72 (45.28) |
| Coinfection |  |  |
| Yes | 6 (5.26) | 17 (10.69) |
| No | 89 (78.07) | 114 (71.70) |
| Unknown | 19 (16.67) | 28 (17.61) |
| TLC Enrollment Date |  |  |
| Oct 2021–Mar 2022 | 14 (12.28) | 1 (0.63) |
| Apr–Sept 2022 | 53 (46.49) | 61 (38.36) |
| Oct 2022–Mar 2023 | 20 (17.54) | 31 (19.50) |
| Apr–Sept 2023 | 15 (13.16) | 12 (7.55) |
| Oct 2023–Mar 2024 | 12 (10.53) | 54 (34.56) |

Abbreviations: HCV, hepatitis C virus; OAT, opioid agonist therapy; IVDU, intravenous drug use; DAA, direct acting antiviral; SVR, sustained virologic response (i.e., viral cure); n, number

^1^Active IVDU, if noted by clinician within 3 months of treatment workup
